# Influence of Nordic walking with poles with an integrated resistance shock absorber on carbohydrate and lipid metabolic indices and white blood cell subpopulations in postmenopausal women

**DOI:** 10.7717/peerj.13643

**Published:** 2022-06-30

**Authors:** Anna Huta-Osiecka, Krystian Wochna, Rafał Stemplewski, Katarzyna Marciniak, Tomasz Podgórski, Zbigniew Kasprzak, Piotr Leszczyński, Alicja Nowak

**Affiliations:** 1 Department of Hygiene, Poznan University of Physical Education, Poznań, Poland; 2 Laboratory of Swimming and Water Lifesaving, Poznan University of Physical Education, Poznań, Poland; 3 Department of Physical Activity Sciences and Health Promotion, Poznan University of Physical Education, Poznań, Poland; 4 Department of Physiology and Biochemistry, Poznan University of Physical Education, Poznań, Poland; 5 Department of Rheumatology, Rehabilitation and Internal Medicine, Poznan University of Medical Sciences, Poznań, Poland

**Keywords:** Nordic walking, Physical activity, Insulin sensitivity, Lipid profile, Vitamin D, Postmenopausal women

## Abstract

**Background:**

Regular and individualised physical activities have been shown to prevent adverse changes associated with the aging process. The main purpose of this study was to evaluate changes in carbohydrate and lipid metabolism and white blood cell (WBC) subpopulations in postmenopausal women participating in Nordic walking (NW) training and to compare the use of poles with an integrated resistance shock absorber (RSA) with the use of classic poles.

**Materials & Methods:**

A total of 23 postmenopausal women participated in a 8-week programme of systematic physical activity between February and April. Before and after the training programme, somatic features and serum concentrations of 25-hydroxyvitamin D, glucose, and insulin, were assessed, as well as lipid profile and WBC count and its specific subpopulations.

**Results:**

Analysis of differences in somatic features and biochemical indices before and after training in the group of women who used RSA poles showed significant decreases in fat mass content (*p* < 0.05), insulin (*p* < 0.05), homeostatic model assessment of insulin resistance (*p* < 0.05), triglycerides (*p* < 0.05), total cholesterol (*p* < 0.05) and monocytes (*p* ≤ 0.01). In the group of women who used classic poles (NW), there was a significant decrease in WBC (*p* ≤ 0.01), lymphocytes (*p* < 0.05), monocytes (*p* ≤ 0.01) and granulocytes (*p* < 0.05).

**Conclusion:**

Increasing the training load through the use of RSA poles resulted in greater changes in carbohydrate and lipid metabolic indices compared to the use of classic NW poles. In turn, the more significant effect on WBC and its specific subpopulations count in the NW, compared to the RSA training programme, may indicate that specificity of training load is an important factor in modifying the immune system response.

## Introduction

During the postmenopausal period women often experience a number of hormonal and metabolic changes that can adversely affect their organisms (Stachowiak, Pertyński & Pertyńska-Marczewska, 2015). However, research has shown that regular physical activity of adequate intensity is an important factor modifying the functioning of most metabolic pathaways and promotes the maintenance of good health (Khalafi, Malandish & Rosenkranz, 2021; Moreira et al., 2014; Sternfeld & Dugan, 2011). Furthermore, individualised and regular physical activities have been shown to prevent adverse changes associated with the aging process (Wang et al., 2020; Woods et al., 2012).

Nordic walking (NW) is a physical activity that is popular, safe, and easily accessible (Bullo et al., 2018). NW is a marching activity with use of poles adapted from cross-country skiing. Using poles enables to engage muscles that are not used during normal walking (Kocur & Wilk, 2006). Among the many different forms of physical training, NW is classified as an aerobic activity in which the whole body is engaged, promoting improvements in physiological parameters and muscle strength and fitness (Pérez-Soriano et al., 2014). An additional advantage of this form of training is that the exercise is performed outdoors, which may contribute to beneficial metabolic effects by increasing vitamin D concentrations in the body (Nowak et al., 2020). Previous studies pointed out the metabolic and anti-inflammatory effects of vitamin D and the relationship between serum 25(OH)D concentrations and subpopulations of WBC has been documented (Mousa et al., 2020).

Pérez-Soriano et al. (2014) found that NW differs from conventional walking in its effects on the musculoskeletal system; it is more stable and can be considered an intermediate mode between walking and running; higher locomotor speeds in comparison to walking result in increased physiological loads, without increasing the subjective perception of effort. Results of a systematic review showed positive effects of NW programmes on anthropometric parameters, body composition, cardiovascular parameters, and glucose tolerance in overweight and obese people (Gobbo et al., 2019). In a study comparing the effects of NW and conventional walking in middle-aged men and women, Muollo et al. (2019) found that NW resulted in more beneficial and faster changes in parameters such as body mass index (BMI), total body fat, android fat, and leg fat, and improved physical performance to a greater extent, compared to walking. Furthermore, in another study, based on a comparison of 6 weeks of NW training with regular walking in postmenopausal women over 55 years old, Cebula et al. (2020) found that for the same speed, NW generated higher energy expenditure than regular walking (without poles). Thus, NW may be a primary and more effective tool than walking for counteracting overweight and obesity in middle-aged adults.

A new form of NW utilises poles with an integrated resistance shock absorber (RSA). In this type of physical activity, the poles used for walking are modified. The premise of the RSA pole design is to increase the load on the upper body by working with resistance (Marciniak et al., 2020). Marciniak et al. (2020) suggested that participants taking part in training with this type of pole had to perform additional work with their upper limbs, thus increasing the overall intensity of the exercise, in comparison to the classical form of NW.

A number of authors have demonstrated the effectiveness of NW as a systematic physical activity through, among others, analysis of lipid and carbohydrate metabolic indices (Hagner-Derengowska et al., 2015a; Hagner-Derengowska et al., 2015b; Prusik et al., 2018; Witkowska et al., 2021). In the review article on physical activity in people with type 2 diabetes, Pesta et al. (2017) suggested that current exercise recommendations to improve metabolic processes should pointed out a synthesis of higher-intensity resistance exercise and lower-intensity resistance training or endurance training. In a study in overweight/obese postmenopausal women, Johannsen et al. (2012) observed a reduction in total WBC and neutrophil counts after an aerobic exercise program in a dose-dependent manner. Taking into account the fact that the degree of modification of metabolic and inflammatory processes in the body depends on the type of training load, the main purpose of this study was to evaluate changes in carbohydrate and lipid metabolism indices and white blood cell (WBC) subpopulations in postmenopausal women participating in training with the use of RSA poles, compared with NW training with the use of classic poles. An additional aim was to evaluate the response of these indices to systematic training with regard to body fat content and 25-hydroxyvitamin D (25(OH)D) concentrations.

## Material and Methods

### Participants and the study protocol

A total of 40 postmenopausal women were enrolled in the study. Women were randomly assigned to two groups according to use the type of poles—classic NW or RSA poles. Randomization was conducted as a simple blind random assignment using a computerized list. This allocation was performed by a person not involved in the conduct of the study. A questionnaire was used to obtain information on lifestyle, diseases, drugs and supplements used, and frequency of fish consumption. Subjects who used hormone replacement therapy or medication modifying lipid metabolism, who declared the presence of diabetes or liver disease, or who had stayed abroad in countries with high levels of sunlight during the two weeks preceding the study were excluded from further stages. Subjects who did not adhere to the study protocol by poor attendance at marching training or declared regular participation in other physical activity were also excluded. Finally, 23 women (NW: *n* = 15, RSA: *n* = 8) aged 66 ± 3.65 years were eligible for the research analysis. Subjects participating in the study declared that they had not previously taken part in organised Nordic walking classes.

Prior to the study, the purpose and method of the study were explained to all subjects, and all participants voluntarily consented to the study in writing. The study was approved by the Bioethics Committee of Karol Marcinkowski Medical University in Poznan (code no. 1041/18 and 245/19).

The study was conducted between winter and spring (February–April), and the women participated in the training programme during this period. Before (1st term of measurement) and after (2nd term of measurement) the training programme, somatic features, serum concentrations of selected indices of carbohydrate and lipid metabolism and the vitamin D metabolite (25(OH)D), and WBC count and its specific subpopulations were assessed.

### Training programme

The training programme lasted 8 weeks, with training sessions held twice a week, for a total of 16 sessions. Women were assigned to two groups based on the type of poles used: classic poles (NW group) and RSA poles with 4 kg resistance strength (RSA group). RSA poles (Slimline BungyPump, Sport Progress International AB, Sweden) have a built-in shock absorber with a total length of 20 cm; marching with the RSA poles therefore leads to different positioning of the upper limbs, in comparison with classic NW poles. On pressing the RSA pole, muscles perform additional work to overcome the resistance of the elastic shock absorber. Pressing the shock absorber changes the length of the pole, which, when shortened by the maximum amount, is the same length as classic NW poles. Releasing the pressure causes the stick to deform to its original length with equal force, potentially causing sensations of altered body balance (Marciniak et al., 2021).

All women (NW and RSA groups) took part in the training at the same time. The training was always conducted by the same NW instructor (Polish Nordic Walking Federation–qualified).

Each training session began with a warm-up that lasted 10–15 min. After each half of the planned distance (approximately 1.7–2.2 km, at a pace of around 1 km per 10 min), participants performed strength exercises and balance training (15 min). Stretching exercises then took place at the end of the planned distance training (15 min). During the sessions, the walking distance was gradually increased from 3.5 to 4.5 km, and the number of exercises performed was increased from 8 to 12 repetitions. Exercise intensity corresponded to 50% heart rate reserve (HRR) during exercise sessions 1–8, while in sessions 9–16, intensity was increased to 65–70% HRR, measured using a heart rate monitor (Polar Electro Oy, Kernpele, Finland). A minimum required attendance of 13 training sessions (80%) was adopted.

Before the intervention, participants were familiarised with the equipment and trained in the correct marching techniques during a 60-minute tutorial session. The training took place in a city park; the subjects walked along the inner lanes of the park, on varied ground. The length of the route was measured using the Endomondo application (Marciniak et al., 2020).

### Fat mass measurement

Fat mass was measured using dual X-ray absorptiometry (DXA) on the whole body. DXA measurements were acquired using a Lunar Prodigy Advance densitometer (General Electric, USA). All measurements were performed by the same technician, using the same instrument. Quality control for the DXA scanner was performed according to the manufacturer’s recommendations, and analyses of the measurements were performed using the integrated software according to the manufacturer’s recommendations.

Height and weight were measured using a certified Radwag (Radom, Poland) device with an accuracy of 0.5 cm. Body mass index (BMI) values were assessed according to the recommendations of the Committee on Diet and Health, taking into account the age of the subjects (Babiarczyk & Turbiarz, 2012).

### Biochemical analysis

Blood was collected from the ulnar vein between 7:30 and 9:30 am (after participants had fasted overnight) and centrifuged to obtain serum for biochemical analysis. Blood serum was stored at −70 °C until biochemical analyses were performed. Biochemical analysis were performed as previously described in Huta-Osiecka et al. (2021). Serum 25(OH)D concentration was determined by chemiluminescent immunoassay (CLIA), using the LIAISON® 25 OH Vitamin D TOTAL Assay (DiaSorin Inc, Saluggia, Italy), with sensitivity 4 ng/ml. The concentrations of glucose and lipid profile (TC, total cholesterol; TG, triglycerides; HDL-C, high density lipoprotein cholesterol; LDL-C, low density lipoprotein cholesterol) were determined using an automatic biochemical analyser (ACCENT 220S; Cormay, Warsaw, Poland) and dedicated enzymatic tests supplied by Cormay (Warsaw, Poland). Sensitivity of tests was 0.41 mg/dl, 1.95 mg/dl, 1.4 mg/dl, 1.1 mg/dl, and 3.9 mg/dl, respectively. Insulin concentration was determined by immunoenzymatic ELISA (DRG Instruments GmbH, Marburg, Germany), with sensitivity 1.76 µIU/ml. Spectrophotometric measurements for the ELISA test were made using a multi-mode microplate reader (Synergy 2 SIAFRT, BioTek, Winooski, VT, USA). The insulin resistance index (HOMA- IR, Homeostatic Model Assessment) was calculated using the formula of Matthews et al. (1985):

\begin{eqnarray*}\text{HOMA-IR}=(\text{Insulin}~[\mathrm{IU/ml}]~\times ~\text{Glucose}~[\mathrm{mmol/l}])/22.5. \end{eqnarray*}

For determination of WBC and selected subpopulation counts (lymphocytes (LYM), monocytes (MON), and granulocytes (GRA)), blood was collected using S-Monovette tubes (Sarstedt, Germany) containing K2-EDTA (EDTA dipotassium salt) as anticoagulant. Haematological measurements were carried out using the 20-parametric automated haematology analyser Mythic® 18 (Orphée, Geneva, Switzerland).

### Statistical methods

Data were collected as previously described in Wochna et al. (2019) and are presented as mean, standard deviation (SD), median and interquartile range. Normality of distribution was verified using the Shapiro–Wilk test. The *t*-test and Mann–Whitney U test were employed for normally and non-normally distributed variables, respectively, to evaluate the significance of differences between groups. The *t*-test and Wilcoxon test were used for normally and non-normally distributed variables, respectively, to evaluate the significance of differences over time (between the first and second times that subjects were tested). A 2 × 2 (group × time interaction) repeated-measures ANOVA was used to evaluate the influence of the training programme on the assessed indices (changes across time within each group). Pearson analysis for normally distributed variables and Spearman’s rank analysis for non-normally distributed variables were used to calculate correlation coefficients. Statistical significance was set at an alpha of 0.05 for all statistical procedures. Statistical analysis of results was performed using Dell Statistica data analysis software (version 13, software.dell.com; Dell Inc., Round Rock, TX, USA).

## Results

Table 1 presents descriptive statistics of somatic features, metabolic indices, and counts of WBC subpopulations in the group of subjects (*n* = 23) measured before and after training (1st and 2nd measurements). Comparative analysis of these parameters before and after training revealed significant changes in body mass (*p* = 0.0153), BMI (*p* = 0.0099), fat mass (%; *p* = 0. 0169), fat mass (kg; *p* = 0.0371), insulin (*p* = 0.0036), HOMA-IR (*p* = 0.0101), TG (*p* = 0.0455), TC (*p* = 0.0101), LYM (*p* = 0.0055), MON (*p* < 0.0001), GRA (*p* = 0.0152), and WBC (*p* = 0.0001). No significant changes were noted for other variables.

**Table 1:** Somatic characteristics, biochemical indices and WBC and its specific subpopulations count in two study measurements (before and after training programme) for the entire group of women (*n* = 23).

Parameters	Assessment at baseline (term I)	Assessment at the end (term II)
Body mass (kg)	68.09 ± 9.60	68.70 ± 9.59
	67.2 (62.4–74.8)	66.6 (62.8–76.2)^*^
BMI (kg/m^2^)	26.22 ± 3.38	26.47 ± 3.31
	25.5 (23.3–28.7)	25.8 (23.6–28.9)^**^
Fat mass (kg)	27.11 ± 6.19	26.59 ± 6.11
	26.1 (22.7–31.0)	25.9 (21.8–30.6)^*^
Fat mass (%)	40.62 ± 4.63	39.98 ± 4.41
	40.3 (37.0–43.7)	40.8 (36.1–43.2)^*^
25(OH)D (ng/ml)	27.01 ± 11.27	27.28 ± 10.82
	25.9 (16.1–32.5)	27.3 (17.8–37.0)
Glucose (mmol/l)	5.21 ± 0.55	5.28 ± 0.55
	5.2 (4.8–5.6)	5.2 (4.8–5.7)
Insulin (µIU/ml)	15.28 ± 6.51	12.13 ± 6.30
	16.4 (9.5–21.3)	10.2 (7.7–17.1)^**^
HOMA-IR	3.56 ± 1.62	2.90 ± 1.63
	3.6 (2.2–5.0)	2.5 (1.5–4.3)^*^
TC (mg/dl)	209.78 ± 36.51	195.70 ± 41.76
	204 (189–240)	182 (167–223)^*^
TG (mg/dl)	101.0 ± 59.77	91.26 ± 48.43
	87 (64–121)	81 (59–108)^*^
HDL-C (mg/dl)	63.71 ± 12.32	62.67 ± 11.39
	64.0 (56.8–71.9)	63.3 (57–70)
LDL-C (mg/dl)	139.27 ± 39.76	138.87 ± 43.98
	128.8 (107.5–165.1)	130.3 (103.9–178.4)
WBC (10^9^/l)	6.35 ± 1.37	5.42 ± 1.26
	6.3 (4.9–7.2)	5.3 (4.6–6.0)^**^
LYM (10^9^/l)	2.1 ± 0.56	1.87 ± 0.40
	2.0 (1.7–2.4)	1.8 (1.5–2.3)^**^
MON (10^9^/l)	0.45 ± 0.12	0.27 ± 0.07
	0.4 (0.4–0.5)	0.3 (0.2–0.3)^**^
GRA (10^9^/l)	3.81 ± 1.12	3.27 ± 0.99
	3.7 (2.8–4.7)	3.1 (2.8–3.5)^*^

**Notes.**

Results are expressed as mean (SD); median (interquartile range).

An asterisk (*) indicate *p* < 0.054, two asterisks (**) indicate *p* < 0.01; significant differences between the first and second terms of the study.

BMI body mass index 25(OH)D 25-hydroxyvitamin D HOMA-IR homeostatic model assessment of insulin resistance index TC total cholesterol TG triglycerides HDL-C high density lipoprotein cholesterol LDL-C low density lipoprotein cholesterol WBC white blood cells LYM lymphocytes MON monocytes GRA granulocytes

Table 2 presents comparative analysis of somatic features, metabolic indices and subpopulations of WBCs (mean values and SD) measured before and after training for groups of women divided by the type of poles used during training (RSA, *n* = 8, and NW, *n* = 15). Comparative analysis of these variables between groups (RSA and NW) before and after training did not show any significant differences. In the RSA group, analysis of changes in somatic features and biochemical indices before and after training revealed significant decreases in fat mass content (%, *p* = 0.0066 and kg, *p* = 0.0142), insulin (*p* = 0.0326), HOMA-IR (*p* = 0.0267), TG (*p* = 0.0117), TC (*p* = 0.0430), and MON (*p* = 0.0038). On the other hand, in the NW group, there were significant increases in body mass (*p* = 0.0049) and BMI (*p* = 0.0047), and decreases in WBC (*p* = 0.0004), LYM (*p* = 0.0271), MON (*p* < 0.0001) and GRA (*p* = 0.0169).

**Table 2:** Somatic characteristics, biochemical indices and WBC and its specific subpopulations counts in the two study terms for two groups of women divided by the type of poles (RSA, *n* = 8 and NW, *n* = 15).

Parameters	Groups	Assessment at baseline (term I)	Assessment at the end (term II)
Body mass (kg)	RSA	69.70 ± 8.33	69.83 ± 8.47
	NW	67.23 ± 10.38	68.09 ± 10.37^**^
BMI (kg/m^2^)	RSA	26.88 ± 3.46	26.96 ± 3.38
	NW	25.87 ± 3.41	26.21 ± 3.36^**^
Fat mass (kg)	RSA	27.31 ± 6.30	26.30 ± 6.07^*^
	NW	27.00 ± 6.35	26.75 ± 6.34
Fat mass (%)	RSA	39.71 ± 5.08	38.69 ± 5.13^**^
	NW	41.10 ± 4.47	40.67 ± 4.00
25(OH)D (ng/ml)	RSA	31.69 ± 14.32	32.24 ± 11.62
	NW	24.51 ± 8.83	24.63 ± 9.74
Glucose (mmol/l)	RSA	5.12 ± 0.76	5.11 ± 0.68
	NW	5.25 ± 0.42	5.36 ± 0.46
Insulin (µIU/ml)	RSA	16.07 ± 5.04	11.66 ± 5.67^*^
	NW	14.86 ± 7.30	12.38 ± 6.79
HOMA-IR	RSA	3.68 ± 1.32	2.75 ± 1.65^*^
	NW	3.50 ± 1.81	2.97 ± 1.68
TC (mg/dl)	RSA	220.38 ± 35.62	197.38 ± 38.76^*^
	NW	204.13 ± 36.91	194.80 ± 44.57
TG (mg/dl)	RSA	100.63 ± 33.38	84.25 ± 28.32^*^
	NW	101.20 ± 71.11	95.00 ± 56.93
HDL-C (mg/dl)	RSA	66.71 ± 15.01	61.86 ± 11.25
	NW	62.11 ± 10.85	63.11 ± 11.83
LDL-C (mg/dl)	RSA	140.26 ± 45.93	141.38 ± 48.60
	NW	138.74 ± 37.81	137.54 ± 43.05
WBC (10^9^/l)	RSA	5.95 ± 1.24	5.50 ± 1.25
	NW	6.57 ± 1.44	5.37 ± 1.31^**^
LYM (10^9^/l)	RSA	2.17 ± 0.29	1.98 ± 0.40
	NW	2.06 ± 0.67	1.81 ± 0.39^*^
MON (10^9^/l)	RSA	0.43 ± 0.13	0.25 ± 0.05^**^
	NW	0.47 ± 0.12	0.29 ± 0.07^**^
GRA (10^9^/l)	RSA	3.35 ± 1.07	3.25 ± 1.08
	NW	4.06 ± 1.10	3.28 ± 0.98^*^

**Notes.**

**Group RSA:** poles with an integrated resistance shock absorber

**Group NW:** classic poles Results are expressed as mean ± SD

An asterisk (*) indicate *p* < 0.05, two asterisks (**) indicate *p* < 0.01; significant differences between the first and second terms of the study.

BMI body mass index 25(OH)D 25-hydroxyvitamin D HOMA-IR homeostatic model assessment of insulin resistance index TC total cholesterol TG triglycerides HDL-C high density lipoprotein cholesterol LDL-C low density lipoprotein cholesterol WBC white blood cells LYM lymphocytes MON monocytes GRA granulocytes

Between groups divided with respect to the type of poles (RSA or NW), we identified a tendency for interactions (group × time) in HDL-C concentrations (F(1,21) = 4.2689; *p* = 0.0514) and WBC count (F(1,21) = 3.7441; *p* = 0.0666) only.

For the whole group (*n* = 23), correlation analysis was carried out to evaluate relationships between several parameters (body mass, BMI, fat mass (% and kg) and 25(OH)D concentrations) assessed before training and changes (Δ) in metabolic indices (glucose, insulin, HOMA-IR, TC, LDL-C, HDL-C, TG) and WBC count and its specific subpopulations (LYM, MON, GRA) assessed after training. The only significant correlation identified was between body mass and Δglucose (*p* = 0.0482).

For the whole group, numerous relationships between changes in specific variables (somatic, metabolic and WBC subpopulations) that occurred after training (Δ) were identified. The following positive correlations were observed: Δbody mass with Δfat mass [%] (*r* = 0.51, *p* = 0.013) and Δfat mass [kg] (*r* = 0.75, *p* < 0.001); ΔBMI with Δfat mass [kg] (*r* = 0.68, *p* < 0.001) and Δfat mass [%] (*r* = 0.48, *p* = 0.022); Δfat mass [%] with ΔLYM (*r* = 0.52, *p* = 0.011); Δinsulin with ΔWBC (*r* = 0.54, *p* = 0.008), ΔGRA (*r* = 0.53, *p* = 0.009), ΔTC (*r* = 0.45, *p* = 0.030) and ΔHDL-C (*r* = 0.43, *p* = 0.043); ΔHOMA-IR with ΔWBC (*r* = 0.056, *p* = 0.006), ΔGRA (*r* = 0.56, *p* = 0.005) and ΔTC (*r* = 0.42, *p* = 0.047); ΔTC with ΔLDL (*r* = 0.68, *p* < 0.001). Negative correlations were observed as follows: Δfat mass [%] with ΔWBC (r = −0.56, *p* = 0.005) and ΔGRA (*r* =  − 0.64, *p* = 0.001); Δfat mass [kg] with ΔWBC (*r* =  − 0.47, *p* = 0.025) and ΔGRA (*r* =  − 0.52, *p* = 0.012); Δinsulin with ΔLYM (*r* =  − 0.46, *p* = 0.26); ΔHOMA-IR with ΔLYM (*r* =  − 0.47, *p* = 0.024); ΔTC with ΔTG (*r* =  − 0.44, *p* = 0.037); ΔWBC with ΔGRA (*r* = 0.90, *p* < 0.001).

There were observed numerous relationships between changes (Δ) in variables that occurred after training for groups divided by the type of poles used (RSA or NW). For the RSA group, the following positive correlations were observed: ΔBMI with Δinsulin (*r* = 0.80, *p* = 0.018), ΔHOMA-IR (*r* = 0.77, *p* = 0.026) and ΔHDL-C (*r* = 0.72, *p* = 0.042); Δfat mass [kg] with Δbody mass (*r* = 0.74, *p* = 0.034); Δinsulin with ΔWBC (*r* = 0.80, *p* = 0.018) and ΔHDL-C (*r* = 0.84, *p* = 0.010); ΔHOMA-IR with ΔWBC (*r* = 0.77, *p* = 0.025) and ΔHDL-C (*r* = 0.83, *p* = 0.012); ΔHDL-C with ΔWBC (*r* = 0.77, *p* = 0.025); ΔLDL-C with ΔLYM (*r* = 0.74, *p* = 0.035) and ΔTC (*r* = 0.95, *p* < 0.001). There were negative correlations for ΔTG with ΔLYM (*r* =  − 0.79, *p* = 0.021), ΔTC (*r* =  − 0.76, *p* = 0.028) and ΔLDL-C (*r* =  − 0.81, *p* = 0.015).

For the NW group of women, positive correlations were observed as follows: Δbody mass with Δtotal fat [kg] (*r* = 0.73, *p* = 0.002) and Δtotal fat [%] (*r* = 0.57, *p* = 0.026); ΔBMI with Δfat mass [kg] (*r* = 0.068, *p* = 0.005) and Δfat mass [%] (*r* = 0.53, *p* = 0.040); Δinsulin with ΔWBC (*r* = 0.68, *p* = 0.005) and ΔGRA (*r* = 0.72, *p* = 0.003); Δglucose with ΔWBC (*r* = 0.51, *p* = 0.050); ΔHOMA-IR with ΔWBC (*r* = 0.67, *p* = 0.006) and ΔGRA (*r* = 0.70, *p* = 0.004). Negative correlations identified were: Δfat mass [%] with ΔWBC (*r* =  − 0.71, *p* = 0.003), ΔGRA (*r* =  − 0.73, *p* = 0.002), Δinsulin (*r* =  − 0.58, *p* = 0.022), and ΔHOMA-IR (*r* =  − 0.57, *p* = 0.028); Δfat mass[kg] with ΔWBC (*r* =  − 0.61, *p* = 0.015), ΔGRA (*r* =  − 0.64, *p* = 0.010), Δinsulin (*r* =  − 0.60, *p* = 0.018), and ΔHOMA-IR (*r* =  − 0.57, *p* = 0.027); ΔLDL-C with Δbody mass (*r* =  − 0.56, *p* = 0.029) and ΔBMI (*r* =  − 0.55, *p* = 0.035).

## Discussion

Many studies have shown that regular exercise promotes the maintenance of good health and is one of the best methods to prevent and treat metabolic diseases. In the present study, after a period of walking training with two types of poles, for the entire group of subjects there was a significant improvement in carbohydrate and lipid metabolism. Reductions in insulin concentrations and HOMA-IR indicates an improvement insulin sensitivity, as well as decreases in lipid metabolic indices (TC and TG) and fat mass [kg and %] were observed. However, comparing the above-mentioned indices in groups divided according to the type of poles used (NW or RSA), despite no significant interaction between RSA and NW, comparative analysis revealed significant differences that occurred after training only in the RSA group. Significant reductions were also observed in WBC and its specific subpopulations counts (LYM, MON, GRA). However, interestingly, significant changes occurred only in the NW group, the opposite of the metabolic indicators.

It is important to note that improvements in carbohydrate and lipid metabolism occurred even though the duration of the training intervention used in this study was relatively short (8 weeks). In a study in postmenopausal women (average age 60 years) Akazawaa et al. (2012) also showed that an 8-week aerobic training period of walking and cycling (average of 47 min, four times a week, 60–75% maximum heart rate) had beneficial effects on weight changes and also led to reduced TG levels and increased HDL-C blood levels.

Regarding NW training, studies have indicated the effectiveness of this specific type of physical activity on metabolic indicators. A study conducted in women with type 2 diabetes showed that a 12-week NW programme (60–90 min, 3 times a week) resulted in significant improvements in anthropometric and metabolic parameters, including reductions in glycosylated haemoglobin (HbA1c) and TG concentrations and an increase in HDL-C concentration (Sentinelli et al., 2015); such significant changes were not observed in the control group, who were also physically active (50 min of any activity, 3 times a week). Ten weeks of NW training were also shown to result in statistically and clinically more significant changes in blood carbohydrate and lipid metabolic markers than Pilates and dietary intervention in overweight and obese women (Hagner-Derengowska et al., 2015a; Hagner-Derengowska et al., 2015b).

NW has been shown to be a more physiologically demanding activity than walking (Cebula et al., 2020), while Muollo et al. (2019) concluded that this form of training can cause more beneficial changes in somatic parameters and increase physical capacity to a greater extent than conventional walking. On the other hand, in a study conducted in postmenopausal women (>55 years) who participated in walking and NW training for 12 weeks (60 min, three times a week); Witkowska et al. (2021) found comparable effects and improvement in blood lipid profile in both study groups (decrease in LDL-C levels in women who performed NW training and decrease in TC and LDL-C levels in women who performed walking training). The purpose of our study was to evaluate whether applying a higher load in RSA pole walking training would have a greater effect on metabolic outcomes than NW. However, we did not observe significant interactions in the response of most metabolic indices to the training programme when comparing groups of women divided by the type of poles used; a tendency in interaction (group x time) occurred only for changes in HDL-C concentration. Nevertheless, it is worth noting that the applied training programme contributed to significant changes in carbohydrate metabolism only in the group of women using RSA poles (decreases in TG, TC, insulin concentration, and HOMA-IR index), for whom changes in fat content were also observed. The results obtained in this study suggest that training loads applied through the use of RSA poles are more effective than classic NW poles. A previous study comparing different training programmes for groups of obese people with and without diabetes (45–65 years) showed that while supervised NW and gym-based programmes were equally effective for improving several parameters (body weight, body composition, muscular flexibility and VO_2_max levels), only NW resulted in significant improvements in concentrations of HbA1c, total and HDL cholesterol (Pippi et al., 2020).

In our study, we measured WBC and its specific subpopulations counts (LYM, MON, GRA). In addition to being immune system cells and non-specific indicators of inflammation, WBC have been reported to be related to carbohydrate metabolism (Lorenzo, Hanley & Haffner, 2014; Vozarova et al., 2002). In their study conducted in a population of nondiabetic Pima Indians, Vozarova et al. (2002) observed that a high WBC count was associated with reduced insulin sensitivity in this group; the authors therefore suggested that chronic activation of the immune system may play a role in the pathogenesis of type 2 diabetes. Furthermore, the Insulin Resistance Atherosclerosis Study, conducted over a period of 5 years in different nondiabetic ethnic groups (56% women; age range 40–69 years), revealed elevated total WBC, neutrophil (NEU) and LYM counts in individuals who were at increased risk of diabetes; LYM count was associated with insulin sensitivity, NEU and MON counts with subclinical inflammation, and total WBC with both insulin sensitivity and subclinical inflammation (Lorenzo, Hanley & Haffner, 2014). In our study, there was a significant decrease in WBC and its specific subpopulation counts (LYM, MON, GRA) after the training period, however, these parameters were reduced within the reference values. The decrease in WBC and GRA counts correlated with the decrease in insulin sensitivity and HOMA-IR observed in the study subjects, confirming the relationship between these indices.

Timmerman et al. (2008) observed that a healthy, physically inactive group of subjects (65- to 80-year-old men and women) had a significantly higher percentage of circulating MON compared with an age-matched physical activity comparison group; they concluded that training by the previously inactive subjects markedly reduced the percentage and count of these proinflammatory cells in the circulatory system. It should be noted, however, that in our study, when women were divided into groups, more significant changes with respect to WBC subpopulation count were noted in the group of women using classic poles (NW), compared with women using RSA poles. Similar changes with respect to WBC were reported in a study on inactive postmenopausal, overweight, and obese women with an average age of 57 years; aerobic training (treadmill walking and semi-recumbent cycle ergometry) for 6 months (50% VO_2_ peak intensity) was observed to reduce WBC and NEU counts in the study group, while the decreases were highest in the group with the highest training load (Johannsen et al., 2012).

The differences between the groups of women (NW and RSA) in the response of WBC subpopulations to the training programme in our study are difficult to explain, but we can conclude that the specificity of the training load is important in modifying the immune system response; however, this requires further research. The results of other studies also confirmed the significant influence of the nature of the training on these indices. For example, Horn et al. (2010) retrospectively analysed the blood test results of elite athletes participating in different endurance sports and showed that more aerobically-oriented sports tended to result in lower WBC and NEU counts, especially when compared to team sports or skill-based sports.

Regarding 25(OH)D concentrations, we did not observe changes in this metabolite during the training period in the study group as a whole (*n* = 23) or when comparing RSA and NW groups. Pilch et al. (2016) found that 6 weeks of NW training in late autumn contributed to lower blood 25(OH)D levels in women older than 55 years. The authors suggested that reduced 25(OH)D levels may have been the result of either decreased vitamin D biosynthesis in the skin (due to decreasing UV intensity during the study period) or vitamin D involvement in muscle metabolism. Therefore, the timing of our study was chosen on the assumption that the intensity of UVB radiation would not change significantly. In the latitude (Poland) where our study took place, this period (February–April) is characterised by low UV intensity (Andersen et al., 2013). We therefore hypothesised that the timing of the study would avoid seasonal changes in serum 25(OH)D concentrations, allowing us to observe its levels and evaluate possible changes in response to exercise load. A number of studies have suggested that physical activity may modify vitamin D levels. For example, Fernandes & Barreto Jr (2017) suggested that physical activity may help to achieve higher vitamin D serum levels in the population, as factors other than sun exposure appeared to be responsible for higher vitamin D levels in more active individuals; however, this phenomenon needs further investigation. In addition, increased vitamin D levels (*p* < 0.0001) were identified in women aged 65–74 years after training outdoors in Nordic walking 3 times a week for 60 min, from April to June; no statistically significant changes were found in the control group (Podsiadło et al., 2021). In light of these results, the authors of this study concluded that physical activity of average intensity, carried out outdoors (with sun exposure), positively affected the level of vitamin D; however, taking other studies into account, they concluded that indoor activity (without direct exposure to sunlight) may also have an positive influence.

In the present study, an additional aim of the analysis was to evaluate the effect of marching training on carbohydrate and lipid metabolic indices in relation to 25(OH)D levels and somatic features (body mass, BMI, fat mass), which were assessed prior to the training programme. A number of previous studies suggested an association between serum 25(OH)D levels and indices of carbohydrate and lipid metabolism (Grimnes et al., 2011; Jungert, Roth & Neuhäuser-Berthold, 2015). Receptors for vitamin D have been identified in pancreatic cells (Christakos et al., 2016), adipose tissue, and the liver (Cimini et al., 2019), indicating that this vitamin is involved in energy metabolism. However, in our previous study, we did not find significant relationships between seasonal changes in 25(OH)D concentration and levels of carbohydrate and lipid metabolic indices in women who did not engage in physical activity during the study period (Huta-Osiecka et al., 2021). In the present study, we also found no association between 25(OH)D concentrations and metabolic indices, as well as no correlation between the changes in metabolic indices (comparing levels before and after training) and 25(OH)D levels either for the whole group or for RSA and NW groups. Thus, we assume that the changes in metabolic indices observed in our study were related to physical activity alone and that serum 25(OH)D levels did not modify these changes.

Studies by other authors indicate a relationship between fat mass content and indices of carbohydrate and lipid metabolism; pathological changes in these indices are particularly observed in overweight and obese individuals (Jabłonowska-Lietz et al., 2017). Women participating in our study were mostly characterised by normal BMI values, with above-normal BMI only found in five people. Zegarra-Lizana et al. (2019) found an association between elevated body fat content (%) and the presence of insulin resistance in a Peruvian population, despite BMI within the normal range. For the whole group of subjects, we did not observe any relationship between body weight, BMI, and fat mass (kg, %) measured prior to the training programme and the magnitude of the changes in metabolic indices due to the training programme. Therefore, we can conclude that the magnitude of changes in these indices was not determined by fat mass content. However, recent studies have revealed a significant relationship between seasonal changes in 25(OH)D concentration and body fat percentage measured at the beginning of the study (autumn period) in postmenopausal women (Huta-Osiecka et al., 2021).

In the current study, we observed that for the whole group, fat mass content (kg and %) was significantly decreased at the end of the training programme, while for groups separated by type of pole, changes in these parameters were only observed in the RSA group. There was a positive correlation between the decrease in fat mass (kg and %) and the decrease in LYM count, while inverse correlations between the decrease in fat mass (kg and %) and the decrease in WBC and GRA counts were observed for the group as a whole. However, it is difficult to explain these differential directions of correlations between changes in body fat and WBC subpopulations. Interestingly, in the NW group, although fat mass did not change significantly after the applied training programme, we observed a significant increase in body weight and BMI, which may indicate a possible increase in lean mass; we did not observe such changes in the group using RSA poles. The significant effect of NW on muscle tissue is confirmed by Micielska et al. (2021) study comparing the effects of NW training with high intensity interval training (HIIT). These authors found that NW was more effective than HIIT at inducing changes in blood exerkine concentrations in elderly people.

The limitation of this study is that it was carried out on a small sample. However, the small size of the group allowed subjects to train at the same time with a single instructor (the same one each time); similar training loads were thus applied to all subjects, with the exception of differences in intensity resulting from the use of two types of poles. In this study we did not analyse the influence of diet and supplements on the indices being studied (especially insulin and lipid indices concentrations). On the other hand, the strength of this study was that it excluded participants who were taking medication which could have affected the data, therefore limiting potential confounding factors.

## Conclusion

A short marching training programme contributed to an improved profile of carbohydrate and lipid metabolic indices in postmenopausal women. These effects were not dependent on 25(OH)D levels or body fat content. Increasing the training load through the use of RSA poles resulted in greater changes in the aforementioned indices compared to classic NW poles. However, in the case of WBC subpopulations, significant changes occurred only in the group of women using NW poles, which may indicate that training load is important in modifying the immune system response; this finding may be the subject of further research.

## Supplemental Information

10.7717/peerj.13643/supp-1 Data S1 Raw data

## Additional Information and Declarations

### Data Availability

The following information was supplied regarding data availability: The raw measurements are available in the Supplementary File.
